# Modulation of Long-Term Potentiation of Cortico-Amygdala Synaptic Responses and Auditory Fear Memory by Dietary Polyunsaturated Fatty Acid

**DOI:** 10.3389/fnbeh.2016.00164

**Published:** 2016-08-23

**Authors:** Daisuke Yamada, Keiji Wada, Masayuki Sekiguchi

**Affiliations:** ^1^Department of Degenerative Neurological Diseases, National Institute of Neuroscience, National Center of Neurology and PsychiatryKodaira, Tokyo, Japan; ^2^AMED-CREST, Japan Agency for Medical Research and DevelopmentChiyoda-ku, Tokyo, Japan

**Keywords:** polyunsaturated fatty acid, cannabinoid, auditory fear circuit, cortico-amygdala synapse, synaptic plasticity

## Abstract

Converging evidence suggests that an imbalance of ω3 to ω6 polyunsaturated fatty acid (PUFA) in the brain is involved in mental illnesses such as anxiety disorders. However, the underlying mechanism is unknown. We previously reported that the dietary ratio of ω3 to ω6 PUFA alters this ratio in the brain, and influences contextual fear memory. In addition to behavioral change, enhancement of cannabinoid CB_1_ receptor-mediated short-term synaptic plasticity and facilitation of the agonist sensitivity of CB_1_ receptors have been observed in excitatory synaptic responses in the basolateral nucleus of the amygdala (BLA). However, it is not known whether long-term synaptic plasticity in the amygdala is influenced by the dietary ratio of ω3 to ω6 PUFA. In the present study, we examined long-term potentiation (LTP) of optogenetically-evoked excitatory synaptic responses in synapses between the terminal of the projection from the auditory cortex (ACx) and the pyramidal cells in the lateral nucleus of the amygdala. We found that LTP in this pathway was attenuated in mice fed with a high ω3 to ω6 PUFA ratio diet (0.97), compared with mice fed with a low ω3 to ω6 PUFA ratio diet (0.14). Furthermore, mice in the former condition showed reduced fear responses in an auditory fear conditioning test, compared with mice in the latter condition. In both electrophysiological and behavioral experiments, the effect of a diet with a high ω3 to ω6 PUFA diet ratio was completely blocked by treatment with a CB_1_ receptor antagonist. Furthermore, a significant reduction was observed in cholesterol content, but not in the level of an endogenous CB_1_ receptor agonist, 2-arachidonoylglycerol (2-AG), in brain samples containing the amygdala. These results suggest that the balance of ω3 to ω6 PUFA has an impact on fear memory and cortico-amygdala synaptic plasticity, both in a CB_1_ receptor–dependent manner.

## Introduction

Increasing evidence suggests that the ratio of ω3 to ω6 polyunsaturated fatty acid (PUFA) in the brain may be an important factor in the modulation of emotion. For example, in humans, analyses of PUFA profiles in serum showed higher ω6 levels and lower ω3 levels in depressed subjects (Maes et al., 1996; Kiecolt-Glaser et al., 2007). The ratio of ω6 to ω3 PUFA in red blood cell membranes was found to be significantly higher in patients with social anxiety disorder compared with control subjects (low ω3 and high ω6; Green et al., 2006). In addition, supplementation of ω3 PUFA was shown to reduce anxiety (Yehuda et al., 2005), and prevent the development of post-traumatic stress disorder (Matsuoka et al., 2010).

The relationship between PUFA and emotional behavior has also been documented in rodents. Lifelong deficiency of ω3 PUFA has been found to result in more depression-like behavior in the forced swim test and anxiety-like behaviors in the open field test compared with controls (Lafourcade et al., 2011; Larrieu et al., 2012). We recently reported that, in ω3-sufficient (normal) mice, the ω3 to ω6 PUFA (3:6) ratio in the diet (see “Materials and Methods” Section in detail), but not the amount of ω3 itself, influenced contextual fear memory in a cannabinoid CB_1_ receptor-dependent manner. Our findings also suggested that short-term synaptic plasticity modulated by a CB_1_ receptor (depolarization-induced suppression of excitation) was enhanced by with a high 3:6 ratio diet in the basolateral nucleus of the amygdala (BLA; Yamada et al., 2014). However, our study did not address whether long-term synaptic plasticity is influenced by a high 3:6 ratio diet.

In addition to these behavioral findings, cannabinoid CB_1_ receptor-dependent long-term depression of excitatory synaptic responses was found to be absent in the medial prefrontal cortex and nucleus accumbens of ω3-deficient mice (Lafourcade et al., 2011). This finding suggests that modulation of long-term synaptic plasticity via CB_1_ receptors in relevant brain regions may be involved in the behavioral effects of ω3-PUFA deficiency (Lafourcade et al., 2011). Therefore, investigating the effect of diets with a high 3:6 ratio upon long-term plasticity may be important for elucidating the mechanism underlying the effects of high 3:6 ratio diet upon fear memory.

In the current study, we investigated the effects of a high 3:6 ratio diet on long-term potentiation (LTP) of synaptic transmission involved in conditioned stimuli in auditory fear conditioning. We have chosen the auditory cortico-amygdala synapse, which is one of the principal synapses that transmits information about tone conditioned stimulus (CS) (Romanski and LeDoux, 1992; Romanski et al., 1993; Boatman and Kim, 2006). To isolate this synaptic transmission, channelrhodopsin-2 was expressed in the auditory cortex (ACx) in mice fed with a high 3:6 ratio diet and mice fed with a low 3:6 ratio diet. We found that auditory fear memory was reduced after consumption of with high 3:6 ratio diet in a cannabinoid CB_1_ receptor-dependent manner, and that intake of a high 3:6 ratio diet caused attenuation of LTP in excitatory synaptic transmission at the synapses from the ACx to the lateral nucleus of the amygdala (LA).

## Materials and Methods

### Animals

Male C57BL/6J mice were purchased (CLEA Japan, Tokyo, Japan) at 4–5 weeks of age and fed with a solid standard mouse diet (CE-2; CLEA Japan) for 1 week. The mice subsequently received test diets for 6 weeks before the electrophysiological or behavioral experiments, as previously described (Yamada et al., 2014). The mice were housed four per cage under controlled temperature (25 ± 1°C) and lighting (12-h light/dark cycle) conditions. Water was provided *ad libitum*. Animal procedures were in strict accordance with the guidelines of the National Institute of Neuroscience, National Center of Neurology and Psychiatry (Tokyo, Japan), and were approved by the Institutional Animal Investigation Committee.

### Diets

We used two diets, which were prepared as previously reported (Yamada et al., 2014). Briefly, we prepared a control diet (a low 3:6 ratio diet) and a test diet (a high 3:6 ratio diet). The low 3:6 ratio diet was similar to the standard diet AIN-93 (Reeves et al., 1993), with soybean oil providing the fat component, and had a value of 0.14 in 3:6 ratio. The high 3:6 ratio diet was prepared by replacing soybean oil with krill oil (rich in ω3 PUFAs), and had a value of 0.97 in 3:6 ratio. The content of ω3 PUFA was 0.44 g/100 g diet in the low 3:6 ratio diet, and was 1.32 g/100 g diet in the high 3:6 ratio diet. Krill oil was prepared from Antarctic krill. The oil was mixed with the other chow components before solidification. The total fat content did not differ between the diets.

### Auditory Fear Conditioning

After the 6-week feeding period, mice were subjected to an auditory fear conditioning test. On day 1, mice were fear conditioned with two pairings of a tone CS (10 kHz, 70 dB, 30 s) and foot shock (unconditioned stimulus [US]; 0.5 mA, 2 s) co-terminated with a tone CS in context A (20 × 20 × 34 cm, white wall and grid floor; Muromachi Kikai, Tokyo, Japan). One hour later, mice were re-exposed to 2 CSs to check the short-term memory (STM). Twenty-four hours after the conditioning, mice were re-exposed to 10 CSs with a 1-min interval in context B (20 × 20 × 34 cm, black wall and bedding on the floor) to check long-term memory (LTM) retrieval and extinction. Forty-eight hours after conditioning, mice were again re-exposed to four CSs for the test. The extent of fear memory was examined by measuring freezing responses (immobility other than respiratory movement). The freezing response was expressed as the percentage of time that the mouse spent freezing during CS presentations (30 s each, 1-min interval), and was analyzed by a well-trained experimenter. In the pharmacological experiment, mice were subcutaneously administered the CB_1_ receptor antagonist rimonabant (RIM; SR141716A, 3.0 mg/kg) or vehicle (VEH) 1 h before conditioning.

### Stereotaxic Surgery and Virus Injection

Mice were anesthetized by intraperitoneal injection of ketamine (100 mg/kg) and xylazine (30 mg/kg). Adeno-associated virus serotype 2 (AAV2) encoding a fusion protein of channelrhodopsin-2 and enhanced yellow fluorescent protein (ChR2-EYFP) under the CaMKIIα promoter (AAV2-CaMKIIα-ChR2[H134R]-EYFP, purchased from the University of North Carolina Vector Core), was stereotaxically infused into the ACx (anteroposterior, −2.92 mm; lateral, ±4.00 mm; ventral, −2.75 mm, 0.5 μL per side) at a rate of 0.05 μL/min. Four to five weeks after virus injection, mice underwent the electrophysiological experiments.

### Electrophysiology

Whole-cell patch-clamp recordings from pyramidal neurons in the LA were performed as described previously (Zushida et al., 2007; Yamada et al., 2012, 2014). Mice were anesthetized with halothane and the brain was quickly removed. Coronal brain slices (300 μm thick), which contained the amygdala, were prepared using a linear slicer Pro 7 (Dosaka EM Co., Ltd., Kyoto, Japan) in artificial cerebrospinal fluid (aCSF; containing [in mM] 125 NaCl, 4.4 KCl, 1.5 MgSO_4_, 1.0 NaH_2_PO_4_, 26 NaHCO_3_, 10 glucose, 2.5 CaCl_2_), pH 7.4, 290–300 mOsm/L. The slices were maintained at 37°C for at least 30 min before recordings in aCSF continuously bubbled with 95% O_2_/5% CO_2_. Each slice was then transferred to the recording chamber and perfused (3 mL/min) with aCSF maintained at 28–32°C (the variability of temperature of aCSF in the recording chamber was constant across experimental groups). Patch electrodes (resistance 4–7 MΩ) were filled with a solution containing the following: (in mM) 132 K-gluconate, 3 KCl, 10 HEPES, 0.5 EGTA, 1 MgCl_2_, 12 sodium phosphocreatine, 3 ATP magnesium salt, 0.5 GTP, pH 7.4, with KOH, 285–290 mOsm/L. The electrophysiological signal was amplified and filtered at 5 kHz using a MultiClamp 700B patch-clamp amplifier (Axon Instruments, Union City, CA, USA). Data were digitized at 50 kHz and acquired using Clampex software (version 9.2, Axon Instruments). Recordings were performed using the voltage-clamp mode. The access resistance, which was frequently checked during recording, was between 11 and 32 MΩ. Cells with a large drift (±20%) in resistance were excluded from the analysis.

Recordings were taken from pyramidal-shaped principal neurons, which were surrounded by ChR2-EYFP positive fibers in LA. To activate ChR2, blue light (465 nm) was delivered (duration = 0.1–1.0 ms) to the recorded neuron through a 63× objective lens (Leica Microsystems, Wetzlar, Germany), using a LED lamp and its driver (BrainVision, Tokyo, Japan). The intensity of the light was set at 25–50% of the maximal synaptic response amplitude (1.8–70.7 × 10^−7^ J under the objective lens). To isolate excitatory synaptic responses, a GABA_A_ receptor antagonist, picrotoxin (100 μM), was included in the aCSF. To check whether the light-evoked excitatory postsynaptic current (LE-EPSC) from ACx to LA was ChR2 dependent, we blocked voltage-gated sodium channels with 1 μM tetrodotoxin (TTX), then additionally applied 1 mM 4-aminopyridine (4-AP) to block the K channels that are critical for the repolarization of the axon. To induce LTP of LE-EPSC, we used an LTP induction protocol that was established in a previous study (Morozov et al., 2011). Briefly, 15 trains of light stimulation (0.1–1.0 ms duration) and synchronous depolarizing current (800 pA, 5 ms duration) were delivered to a postsynaptic cell at 10 Hz under the current-clamp mode (membrane potential of the recorded cell was clamped at −70 mV). This train was repeatedly delivered five times with a 10-s interval. LE-EPSC was monitored for 40 min from 15 s after the end of the last train of the LTP-induction stimulus. The potentiation of LE-EPSC was estimated by normalizing the LE-EPSC amplitude after the induction with a baseline value before the induction. In some experiments, slices were pretreated with RIM (5 μM) prior to the recordings. To check the agonist sensitivity of CB_1_ receptors, the LE-EPSC was recorded every 15 s (at 0.067 Hz) for 5 min before and 10 min after the bath application of the CB_1_ agonist WIN55, 212-2 (WIN; 0.3, 1.0, 3.0, and 10 μM). After the experiments, CNQX (20 μM) and MK-801 (10 μM) were concurrently applied to confirm that the LE-EPSC was glutamatergic.

### Quantification of 2-Arachidonoylglycerol in the Brain

Using a separate group of mice that were not used in the behavioral testing, the content of 2-arachidonoylglycerol (2-AG) in the brain tissue was quantified as previously reported, with a slight modification (Zhang et al., 2010). Immediately after decapitation, brain samples were prepared (Zushida et al., 2007). Briefly, we prepared brain slices including the amygdala (near bregma −1.7 mm level). The delta region lying between the external capsule (mainly consisting of the amygdala) was cut from the slices and saved for quantification of 2-AG. These samples were weighed and placed in glass tubes containing 0.02% trifluoroacetic acid (TFA) and acetonitrile with 40 ng 2-AG-d8, as the internal standard. Tissue was homogenized and centrifuged at 5000 × g for 5 min. Supernatants were transferred to a new tube and the process was repeated one more time before the supernatants were evaporated. The samples were then resuspended in 400 μL acetonitrile and stored at −80°C until use. Samples were analyzed by liquid chromatography mass spectrometry (Agilent Technologies 1200 and Triple Quad LC/MS Agilent Technologies 6410; Agilent Technologies, Santa Clara, CA, USA).

### Measurement of Cholesterol Content in the Brain

We used a separate group of mice that were not used in the behavioral testing to extract total lipids from amygdala-rich brain tissues. These samples were prepared with a similar method to that used for quantification of 2-AG, and cholesterol content was measured using a Wako Cholesterol E kit (Wako Pure Chemical Industry Ltd., Osaka, Japan) according to the manufacturer’s protocol.

### Drugs

RIM was purchased from Cayman Chemicals (Ann Arbor, MI, USA). WIN, picrotoxin, CNQX disodium, and MK-801 hydrogen maleate were purchased from Sigma (St. Louis, MO, USA). 4-AP was purchased from Tokyo Chemical Industry (Tokyo, Japan). For subcutaneous administration, RIM was dissolved in VEH containing 2.5% dimethyl sulfoxide and 1.0% Tween-80 in saline solution. For electrophysiological experiments, all drugs except for WIN were dissolved in aCSF (final concentration): picrotoxin (100 μM), CNQX (20 μM), MK-801 (10 μM), and 4-AP (1 mM). WIN was dissolved in dimethyl sulfoxide at 10 mM and diluted in aCSF at final concentrations (0.3, 1.0, 3.0, and 10 μM) as indicated in the text.

### Statistical Analysis

All data are shown as mean ± SEM. The data were analyzed using one-way analysis of variance (ANOVA) for comparisons among three or more groups. If the ANOVA results were significant, *post hoc* Bonferroni multiple comparisons were performed. Two-tailed unpaired *t*-tests were used for statistical comparisons between two groups. *P* < 0.05 was considered statistically significant.

## Results

### Dietary Ratio of ω3 to ω6 PUFA Influences Auditory Fear Memory

**Figure 1:**
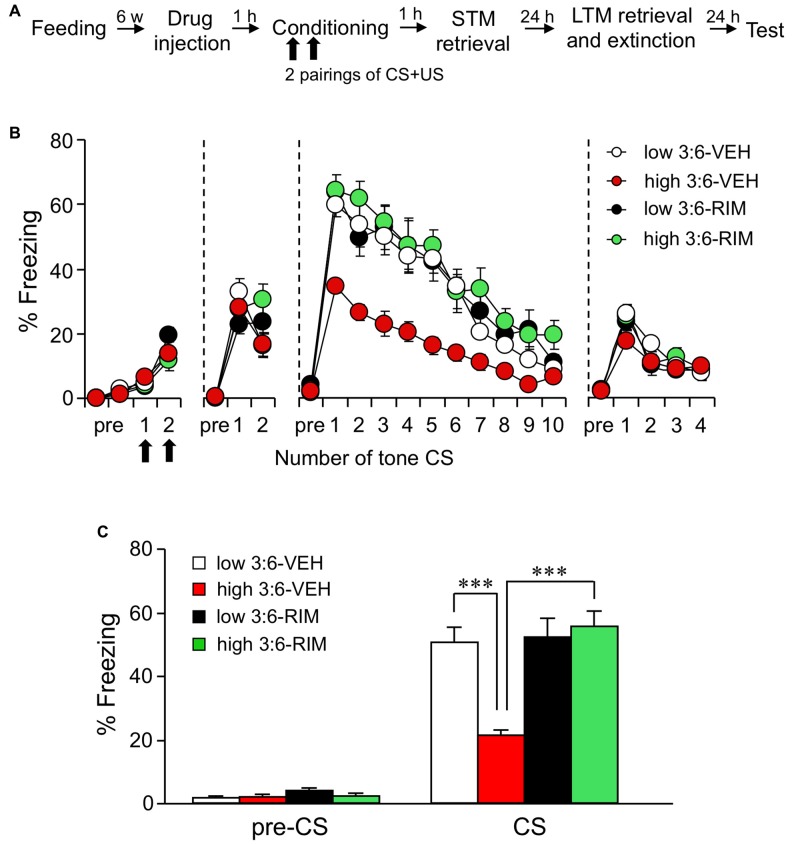
**The ω3 to ω6 polyunsaturated fatty acid (PUFA; 3:6) ratio in the diet influences auditory fear memory. (A)** Experimental schedule of auditory fear conditioning test. **(B)** Time course of the freezing rate of mice in conditioning, short-term memory (STM) test, long-term memory (LTM) retrieval and extinction, and test sessions. **(C)** In the LTM retrieval and extinction session conducted 24 h after conditioning, freezing rate was reduced in mice fed with a high 3:6 ratio diet compared with that in mice fed with a low 3:6 ratio diet. Vehicle (VEH) was injected as the control for rimonabant (RIM; 3 mg/kg) treatment. Administration of RIM 1 h before the conditioning blocked the effect of with a high 3:6 ratio diet on fear memory. *n* = 9 for the low 3:6-VEH group, and *n* = 10 for the other groups. All data are shown as means ± SEM. ***P < 0.001, one-way analysis of variance (ANOVA) and *post hoc* Bonferroni multiple comparison.

We first checked whether auditory fear memory was influenced by 3:6 ratio diet as well as contextual fear memory, based on our previous observation (Yamada et al., 2014). After a 6-week-feeding period, auditory fear conditioning was carried out using a tone as CS and electrical foot shock as US. Then, 1, 24, and 48 h after conditioning, STM test, LTM retrieval and extinction, and test sessions were performed and fear memory was tested by measuring the freezing response to CS alone (Figure 1A). Each mouse was injected subcutaneously with a CB_1_ receptor antagonist RIM or VEH, 60 min before conditioning (*n* = 9 for the low 3:6-VEH group and *n* = 10 for the other groups). Figure 1B shows the time course of changes of the freezing response during conditioning, STM test, LTM retrieval and extinction, and test sessions, respectively. There were no differences in the freezing response among all groups in conditioning (*F*_(3,35)_ = 2.23, *P* = 0.10, one-way ANOVA) and STM test (*F*_(3,35)_ = 0.83, *P* = 0.48, one-way ANOVA) sessions. These results indicate that the memory acquisition was not influenced by a high 3:6 ratio diet. In contrast, in the LTM retrieval and extinction sessions, retrieval of memory was reduced in mice in the high 3:6-VEH group. Freezing during CS was lower in mice in the high 3:6-VEH group compared with that in mice in the low 3:6-VEH group (comparison between white and red circles, Figure 1B). The freezing responses were gradually reduced with the number of CS in all groups, and the reduction rate of the freezing response was similar in mice in the low and high 3:6-VEH groups, suggesting that fear extinction was not affected by diet. Moreover, the reduction of freezing responses in mice in the high 3:6 diet condition was not observed when mice were injected with 3 mg/kg RIM (comparison between red and green circles, Figure 1B), suggesting the involvement of the cannabinoid system in the diet-related difference in freezing response. Figure 1C shows the proportion of time that these mice spent freezing before and during CS presentations in the LTM retrieval and extinction sessions. Statistical analysis confirmed that these effects were significant (*F*_(3,35)_ = 12.47, *P* < 0.0001, one-way ANOVA; *P* < 0.001 for the comparison between the low 3:6-VEH and the high 3:6-VEH groups, *P* < 0.001 for the high 3:6-VEH and the high 3:6-RIM groups, *post hoc* Bonferroni multiple comparison).

### Optogenetic Analysis of Synaptic Responses in Auditory Cortico-Amygdala Synapses

#### Optogenetic Isolation of Synaptic Responses at a Synapse From the ACx to the LA

**Figure 2:**
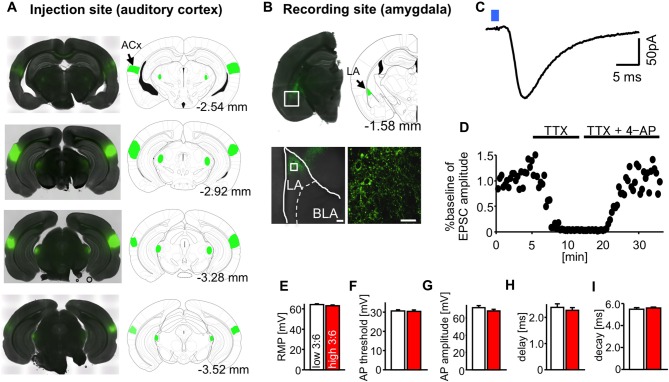
**Optogenetic isolation of auditory cortico-amygdala synapses. (A)** Images showing the expression of channelrhodopsin-2 and enhanced yellow fluorescent protein (ChR2-EYFP; green) in the injection site auditory cortex (ACx), cellular staining; primary auditory cortex, Au1; ventral ACx, AuV; temporal association cortex, TeA). **(B)** Images showing the expression of ChR2-EYFP (green) in the recording site (LA, presynaptic puncta) 4–5 weeks after the injection of adeno-associated virus serotype 2 (AAV2)-ChR2-EYFP into the ACx of mice. Scale: 40 μm. **(C)** A typical light-evoked excitatory postsynaptic current (LE-EPSC) recorded from an LA pyramidal neuron in response to blue light irradiation from a 63× objective lens (blue square, duration of irradiation = 0.5 ms), under whole cell patch clamp recordings at −70 mV. Scale: 5 ms and 50 pA. **(D)** The similarly elicited LE-EPSC was abolished by the perfusion of tetrodotoxin (TTX) and recovered after additional perfusion with 4-AP. **(E–I)** The average values for resting membrane potential (RMP) **(E)**, action potential (AP) threshold **(F)**, AP amplitude **(G)**, synaptic delay **(H)**, and synaptic decay time constant **(I)** in LA neurons in slices from mice fed high and low 3:6 ratio diets. *n* = 9 for all groups.

We used optogenetic methods to record neurotransmission in specific neural pathways in the fear circuit. We chose the auditory cortico-amygdala synapse, which is reported to be important for the processing of tone CS in auditory fear conditioning (Boatman and Kim, 2006). For this purpose, ChR2-EYFP was expressed in the ACx of mice (Figure 2A), and patch-clamp recordings were conducted in LA pyramidal neurons in brain slices from these mice (Figure 2B). In the LA, nerve endings from the ACx that express ChR2 were activated by local blue light irradiation (λ = 465 nm) through a 63× objective lens (light energy, 1.8–70.7 × 10^−7^ J; Figure 2B). The energy of light stimulation was in a similar range to that reported in a previous study observing this pathway (Morozov et al., 2011). We chose pyramidal-shaped principal neurons for recording, which were surrounded by ChR2-EYFP-positive fibers.

Figure 2C shows an example of LE-EPSC. A similar evoked response was completely suppressed by perfusion with TTX (1 μM) and then recovered by further perfusion with 4-AP (1 mM, Figure 2D), suggesting that they were ChR2-evoked synaptic responses. A similar evoked response was abolished by perfusion with an AMPA receptor antagonist CNQX (20 μM, data not shown). The average resting membrane potential (RMP; Figure 2E), action potential (AP) threshold (Figure 2F), AP amplitude (Figure 2G), synaptic delay (Figure 2H), and synaptic decay time constant (Figure 2I) in recorded LA neurons were not significantly affected by diet.

#### CB_1_ Receptor-Dependent Reduction in Plasticity Changes of LE-EPSC in a Synapse from the ACx to the LA

**Figure 3:**
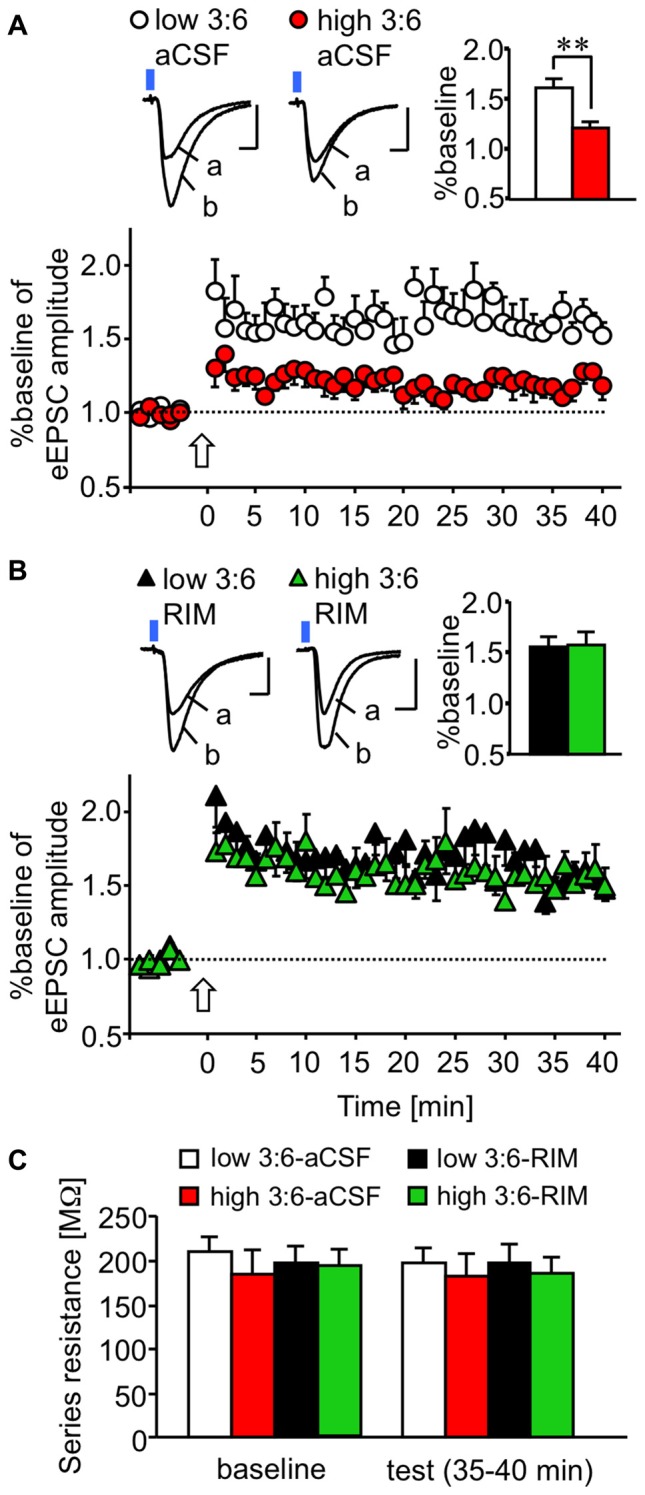
**CB_1_ receptor-dependent reduction in plastic changes (LTP) of excitatory synaptic transmission in a synapse from the ACx to the LA. (A,B)** Time-course of changes in the amplitude of synaptic responses induced by light irradiation, for 5 min before and 40 min after LTP induction in the absence **(A)** or presence **(B)** of bath-applied RIM (for the aCSF groups, nine cells from nine mice fed with a low 3:6 ratio diet and nine cells from eight mice fed with a high 3:6 ratio diet; for the RIM-treated groups, six cells from six mice fed with the low 3:6 ratio diet and six cells from five mice fed with the high 3:6 ratio diet). Above, traces showing representative recordings in individual neurons. Blue square indicates light irradiation, and open arrow indicates LTP induction. Insets are average values of the last 5 min in the recording. **P < 0.01 (one-way ANOVA with *post hoc* Bonferroni multiple comparison). Scale: 5 ms and 50 pA. **(C)** Series resistance in LTP experiments. Series resistance was monitored by injection of voltage step (2 mV) throughout the experiment. Averaged values during baseline (5 min) and last 5 min of the recordings (35–40 min) were compared in each group. White bar: low 3:6 ratio in aCSF, red bar: high 3:6 ratio in aCSF, black bar: low 3:6 ratio in the presence of RIM, green bar: high 3:6 ratio in the presence of RIM. *n* = 9 for all groups.

We examined plasticity changes in LE-EPSC. LTP of the LE-EPSC was induced by pairing trains of light-stimulation and synchronous depolarization of a postsynaptic cell (see “Materials and Methods” Section). Figure 3A shows the time course of changes in the relative amplitude of LE-EPSC (normalized to the value before LTP induction) following treatment with aCSF as a control for RIM-treatment described below. The relative amplitude was higher after LTP induction than before LTP induction in cells from mice in both high and low 3:6 ratio diet groups. This change lasted for at least 40 min after LTP induction. This potentiation in amplitude was weaker in cells from mice fed with a high 3:6 ratio diet (*n* = 9, red circles) compared with mice fed with a low 3:6 ratio diet (*n* = 9, white circles) throughout the experiment. This difference in the extent of LTP between the slices in the two diet conditions was not observed when the slices were treated with RIM (5 μM) prior to recording (*n* = 9 for low 3:6-RIM [black triangles] and *n* = 9 high 3:6-RIM [green triangles] groups; Figure 3B). The relative amplitude values 35–40 min after LTP induction were summed in each condition and statistically analyzed (bar graphs in the far right upper section, Figures 3A,B). The analysis revealed a significant difference between the low 3:6-aCSF and high 3:6-aCSF groups (*F*_(3,26)_ = 4.59, *P* = 0.01 with one-way ANOVA; *P* < 0.01 with *post hoc* Bonferroni comparison; Figure 3A). There was also a significant difference between the high 3:6-aCSF and high 3:6-RIM groups (*P* < 0.05), both determined using *post hoc* Bonferroni multiple comparison tests. Figure 3C shows the mean series resistance during baseline 5 min and last 5 min (35–40 min) in LTP experiments for each experimental condition. There were no substantial changes in resistance in any condition. Taken together, these results suggest that the extent of LTP in LE-EPSC was lower in mice fed with a high 3:6 ratio diet, compared with mice fed with low 3:6 ratio diet, and that the CB_1_ receptor was involved in this difference.

#### Sensitivity of CB_1_ Receptor for its Agonist was Enhanced in the LE-EPSC in a Synapse from the ACx to the LA

**Figure 4:**
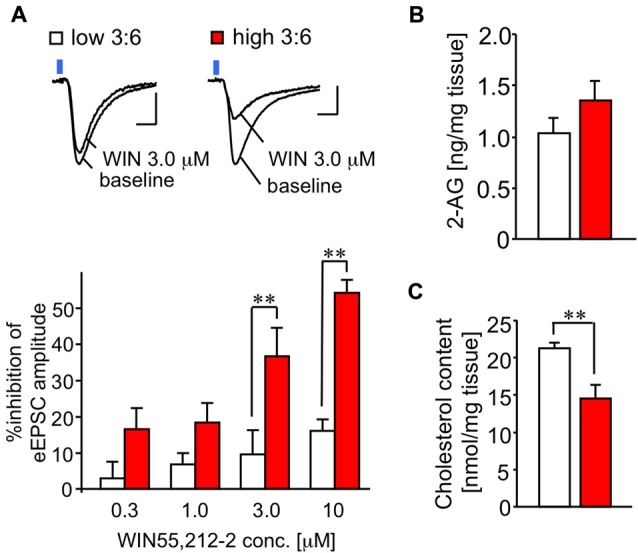
**Sensitivity of CB_1_ receptor for its agonist WIN is enhanced in the LE-EPSC. (A)** Representative recordings in individual neurons before (baseline) and after the application of 3.0 μM WIN (top). Reduction of amplitude of synaptic response by various concentrations of WIN (0.3, 1.0, 3.0, and 10 μM; *n* = 6–7; bottom). Scale: 5 ms and 50 pA. Blue square indicates light irradiation. **(B)** Content of 2-arachidonoylglycerol (2-AG) in the amygdala-rich brain samples (*n* = 5 per group). **(C)** Cholesterol content in amygdala-rich brain slices (*n* = 6 for each). All data are shown as means ± SEM. Scale: 5 ms and 50 pA. **P < 0.01, one-way ANOVA and *post hoc* Bonferroni multiple comparison in **(A)** and Student’s *t*-test in **(C)**.

The difference in the extent of LTP disappeared in the slices treated with a CB_1_ receptor antagonist RIM (Figure 3B). This finding suggests that the activity of the CB_1_ receptor in the synapses we observed may have been enhanced in mice fed with a high 3:6 ratio diet, compared with mice fed with low 3:6 ratio diet. To verify this possibility, we examined the effect of the CB_1_ receptor agonist, WIN, on LE-EPSC. The activation of CB_1_ receptors by their agonists typically results in suppression of the synaptic responses in the amygdala (Azad et al., 2003; Yoshida et al., 2011), although the extent of the suppression effect of WIN on EPSC is reported to be weaker in the LA than in the BLA (Yoshida et al., 2011). In the current results, we found that WIN suppressed the amplitude of LE-EPSC, and this suppression was more pronounced in mice fed with a high 3:6 ratio diet (*n* = 6) than in mice fed with a low 3:6 ratio diet (*n* = 7; Figure 4A). These results suggest that the agonist sensitivity of CB_1_ receptors was enhanced in this synapse by a high 3:6 ratio diet.

#### No Alteration in Endocannabinoid Content in Amygdala-Rich Brain Samples

To test whether the level of the endocannabinoid 2-AG was affected by the diet condition, we quantified 2-AG in the lipid fractions prepared from the brain samples (see “Materials and Methods” Section) of mice fed with both diets. There was no significant difference in 2-AG values (1.02 ± 0.15 ng/mg tissue for low 3:6; 1.44 ± 0.21 ng/mg tissue for high 3:6, *n* = 5 for each group, *t*_(8)_ = 1.83, *P* = 0.10, *t*-test; Figure 4B).

#### Reduction in Cholesterol Content in Amygdala-Rich Brain Samples

Incorporation of ω3 PUFA into the membranes has been shown to decrease membrane-bound cholesterol (Stillwell and Wassall, 2003), which enhances the efficacy of the CB_1_ receptor agonist by attenuating negative allosteric modulation of this receptor by cholesterol (Bari et al., 2005). Therefore, we measured cholesterol content in the brain samples, using a preparation method similar to that used for the quantification of 2-AG. The cholesterol content in the brain samples was significantly lower in mice fed with a high 3:6 ratio diet than in mice fed with a low 3:6 ratio diet (21.29 ± 0.76 nmol/mg tissue for low 3:6; 14.55 ± 1.83 nmol/mg tissue for high 3:6; *t*_(8)_ = 3.40, *P* = 0.009; Figure 4C). This result supports the possibility that attenuation of negative allosteric modulation of the CB_1_ receptor by a high 3:6 ratio diet inducing a decrease of membrane-bound cholesterol is one of the mechanisms underlying the modification of synaptic plasticity in the auditory cortico-amygdala synapses (Yamada et al., 2014).

## Discussion

The current study found that a relatively short-term (6-weeks) intake of a high 3:6 ratio diet causes CB_1_ receptor–dependent attenuation of LTP in excitatory synaptic transmission at synapses that connect the ACx to the LA. Auditory fear memory was also reduced in parallel. To date, the mechanism underlying the alteration of emotion by dietary PUFA has not been fully understood. However, a study by Lafourcade et al. (2011) suggested the involvement of CB_1_ receptor-induced modulation of synaptic plasticity in the brain regions related to emotional behavior, including the medial prefrontal cortex and nucleus accumbens. Lafourcade et al. (2011) reported that ω3 deficiency weakened the long-term depression of excitatory synaptic responses in the prelimbic area of the medial prefrontal cortex and nucleus accumbens, suggesting reduced inhibitory tone on excitatory synaptic transmission associated with emotional behaviors in these regions in mice fed with a ω3-deficient diet. In accord with this finding, the present results showed that a high 3:6 ratio diet (relatively high ω3 PUFA) led to attenuation of LTP of excitatory synaptic responses, suggesting decreased excitatory tone of the neural circuit related to auditory fear memory. Therefore, the modulation of synaptic plasticity to reduce excitatory tone in the relevant brain region may be one of the mechanisms underlying the modulation of emotional behaviors by PUFA.

Several factors should be considered in the interpretation of these results. First, it is problematic to presume that the effect of a high 3:6 ratio diet on LTP was causally related to the reduction of fear memory, because ACx–LA LTP is just one among many forms of plasticity related to fear memory. The thalamo-cortico-amygdala pathway (McDonald, 1998) has been reported as one route through which auditory information is transmitted to LA neurons as CS in auditory fear conditioning (Romanski and LeDoux, 1992; Boatman and Kim, 2006). In addition, it has been suggested that neurons in the dorsal LA respond to both somatosensory (foot shock) and auditory stimuli (Romanski et al., 1993). However, the direct thalamo-amygdala pathway acts as an alternative route (Romanski and LeDoux, 1992) and is necessary for fear conditioning, at least during the early phases of learning (McEchron et al., 1995, 1996; Quirk et al., 1997). Moreover, it has been shown that US-CS association occurs within the central nucleus of the amygdala (Han et al., 2015). Therefore, further studies will be required to clarify whether plasticity in LA neurons projected by the ACx corresponds directly to US-CS association.

Converging evidence suggests that the activation of CB_1_ receptors by excessive synaptic activation triggers feedback inhibition of synaptic transmission (Kano et al., 2009). In accord with these findings, our hypothesis regarding the PUFA balance-induced modification of fear memory is that CB_1_ receptor distribution in the presynaptic site of cortico-amygdala synapses acts to suppress excitatory synaptic transmission via feedback inhibition. This hypothesis is supported by a previous finding that the CB_1_ receptor is distributed in the LA (Yoshida et al., 2011), and the current finding that WIN, a CB_1_ receptor agonist, suppressed LE-EPSC amplitude. Ingestion of a high 3:6 ratio diet may change membrane fluidity at the presynaptic site of cortico-amygdala synapses, inducing the release of the CB_1_ receptor from negative allosteric modification by cholesterol. The current finding that cholesterol level was decreased in brain samples from mice fed with a high 3:6 ratio is consistent with this mechanism.

Taken together with our previous findings (Yamada et al., 2014), the current results suggest that retrieval of both contextual and cued (auditory) fear memory can be reduced by a high 3:6 ratio diet through activation of CB_1_ receptors. In line with these findings, several previous studies have shown that pharmacological activation of CB_1_ receptors attenuates fear memory (Pamplona and Takahashi, 2006; Resstel et al., 2008; Mackowiak et al., 2009; Lisboa et al., 2010). In contrast to the activation of CB_1_ receptors, CB_1_ receptor deficient mice showed impairment of auditory fear extinction without any changes in acquisition and memory retrieval (Marsicano et al., 2002). Thus, it seems that genetic deletion and pharmacological activation have different effects on fear memory.

Furthermore, we quantified the levels of an endocannabinoid, 2-AG, because an increase in 2-AG caused by a high 3:6 ratio diet could underlie an increase in CB_1_ receptor tone in the brain. However, our results showed that 2-AG was not significantly increased in the brain samples from mice fed wit a high 3:6 ratio diet. However, because this result was from bulk brain samples, it remains possible that 2-AG released locally around synapses undergoing excessive activation was increased in mice fed with a high 3:6 ratio diet. It is also possible that CB_1_ receptor expression, or membrane insertion, was increased in mice fed with a high 3:6 ratio diet. Although we cannot exclude the possibility that membrane insertion of CB_1_ receptors is changed by 3:6 ratio diet, we previously found no alteration in the mRNA level of CB_1_ receptors in the brain across high and low 3:6 ratio conditions (Yamada et al., 2014).

In conclusion, the current results revealed that feeding mice with a high 3:6 ratio diet reduced LTP of the synaptic response in auditory cortico-amygdala synapses, accompanied by attenuation of auditory fear memory. The reduction of both LTP and memory were dependent on the cannabinoid CB_1_ receptor.

## Author Contributions

DY and MS designed the experiments. MS supervised the project. DY performed the experiments. DY and MS analyzed the data. DY and MS wrote the manuscript. DY, MS, and KW developed analytical tools. All authors discussed the results and implications and commented on the manuscript.

## Funding

This work was supported by the following grants: KAKENHI (grant number 23500474, 15K06730), Intramural Research Grant for Neurological and Psychiatric Disorders of the National Center of Neurology and Psychiatry (grant numbers 25-1, 27-3; to MS), and a Grant-in-Aid for Scientific Research on Innovative Areas, Foundation of Synapse and Neurocircuit Pathology (to KW), and KAKENHI for young scientists (grant number 15K21653 to DY).

## Conflict of Interest Statement

The authors declare that the research was conducted in the absence of any commercial or financial relationships that could be construed as a potential conflict of interest.
